# Supplementary material for: Urinary Medium-Chained Acyl-Carnitines Sign High Caloric Intake whereas Short-Chained Acyl-Carnitines Sign High -Protein Diet within a High-Fat, Hypercaloric Diet in a Randomized Crossover Design Dietary Trial
Source: Nutrients. 2021 Apr 3;13(4):1191. doi: 10.3390/nu13041191 (PMC8066704; doi:10.3390/nu13041191)

Figure 2

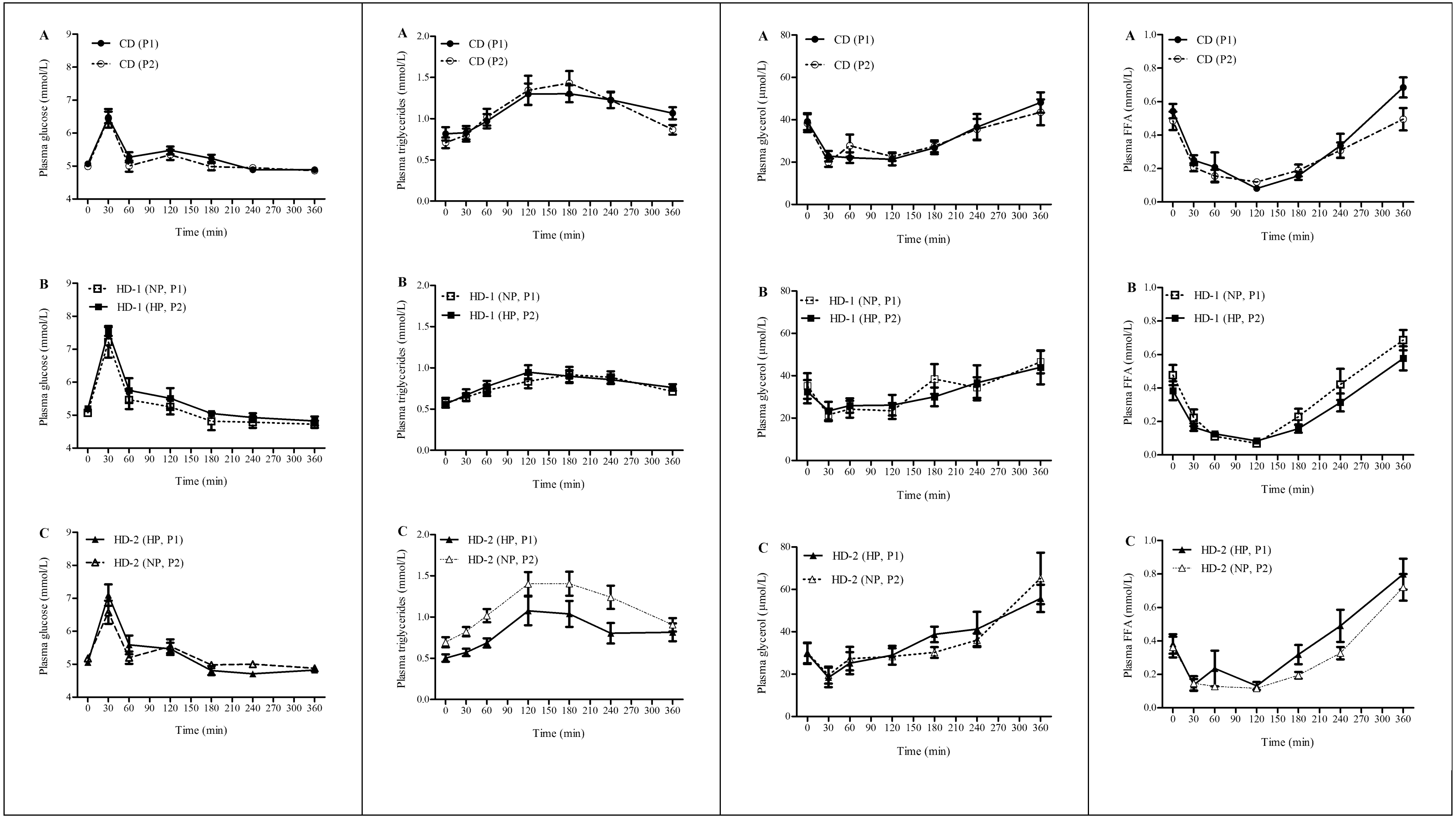

A
CD (P1)
CD (P2)
Plasma glucose (mmol/L)
Time (min)
Plasma triglycerides (mmol/L)
Plasma glycerol (µmol/L)
Plasma FFA (mmol/L)
B
HD-1 (NP, P1)
HD-1 (HP, P2)
C
HD-2 (HP, P1)
HD-2 (NP, P2)

Figure 3

A

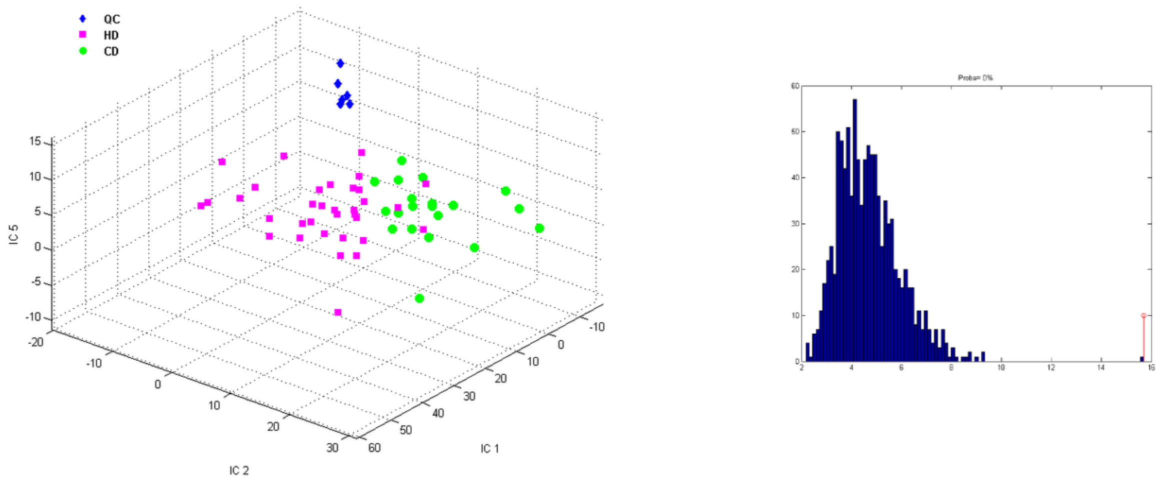
QC
HD
CD
IC 5
IC 2
IC 1
Proba= 0%

B

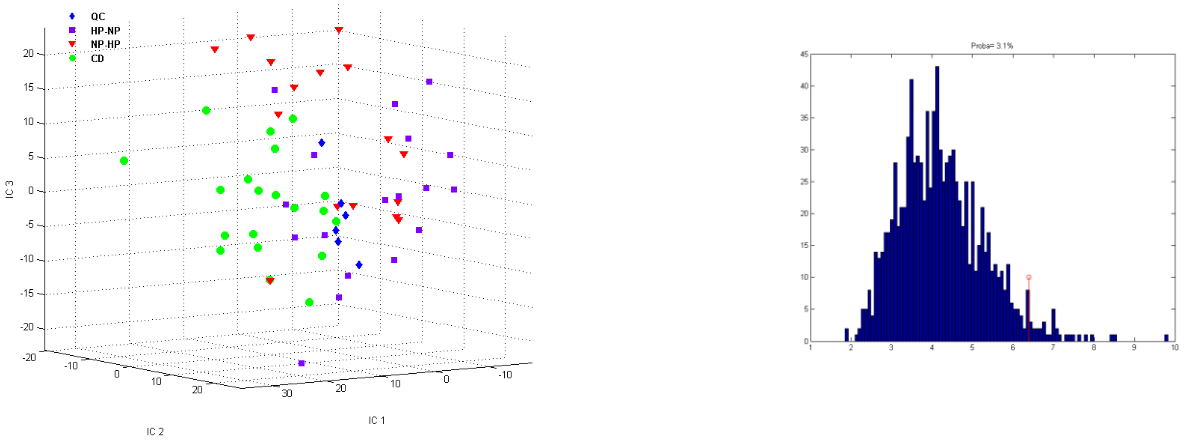
QC
HP-NP
NP-HP
CD
IC 3
IC 2
IC 1
Proba= 3.1%

C

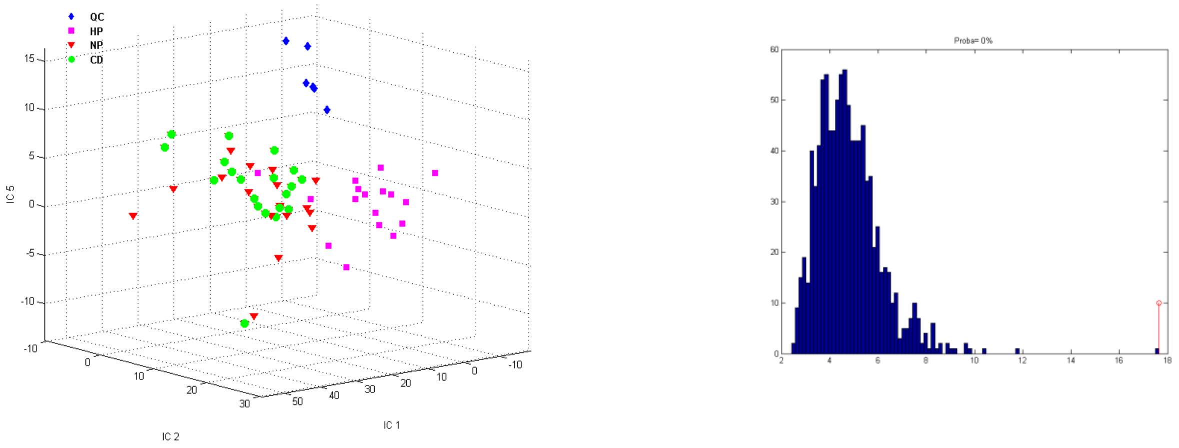
QC
HP
NP
CD
IC 5
IC 2
IC 1
Proba= 0%

Figure 4

A

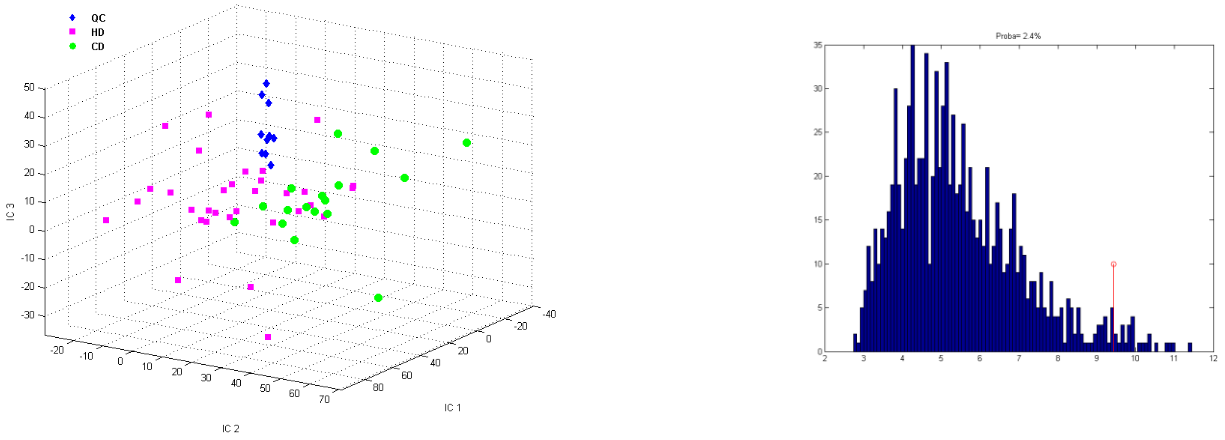
QC
HD
CD
IC 3
IC 2
IC 1
Proba= 2.4%

B

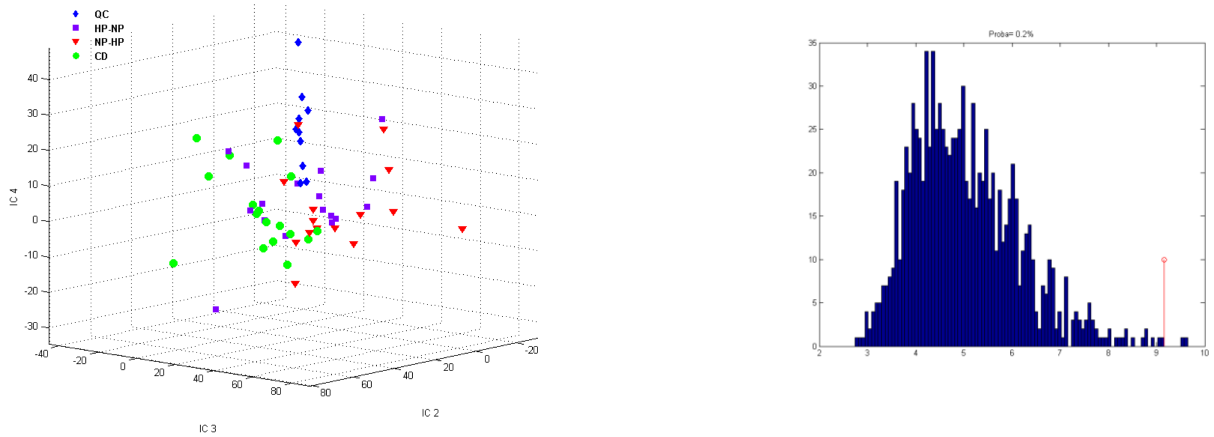
QC
HP-NP
NP-HP
CD
IC 4
IC 3
IC 2
Proba= 0.2%

C

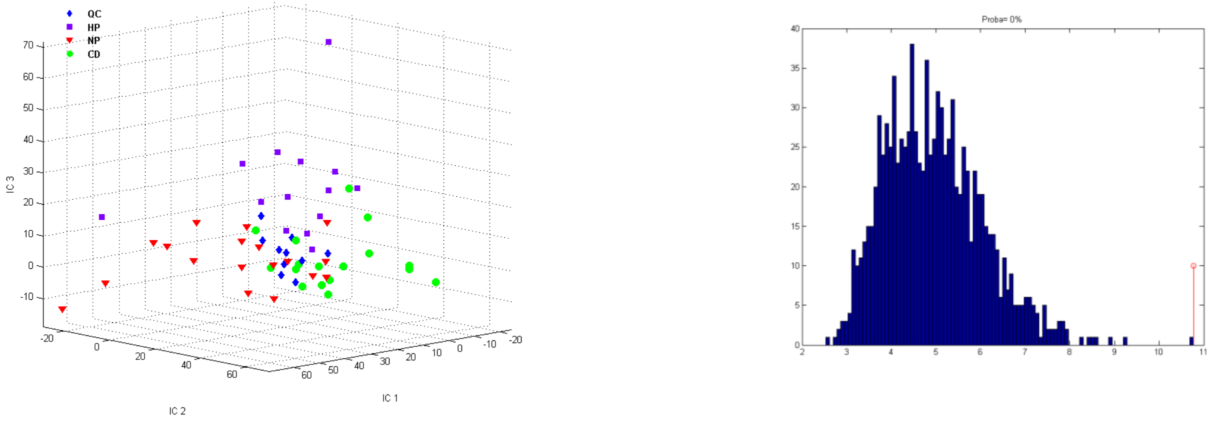
QC
HP
NP
CD
IC 3
IC 2
IC 1
Proba= 0%

Figure 5

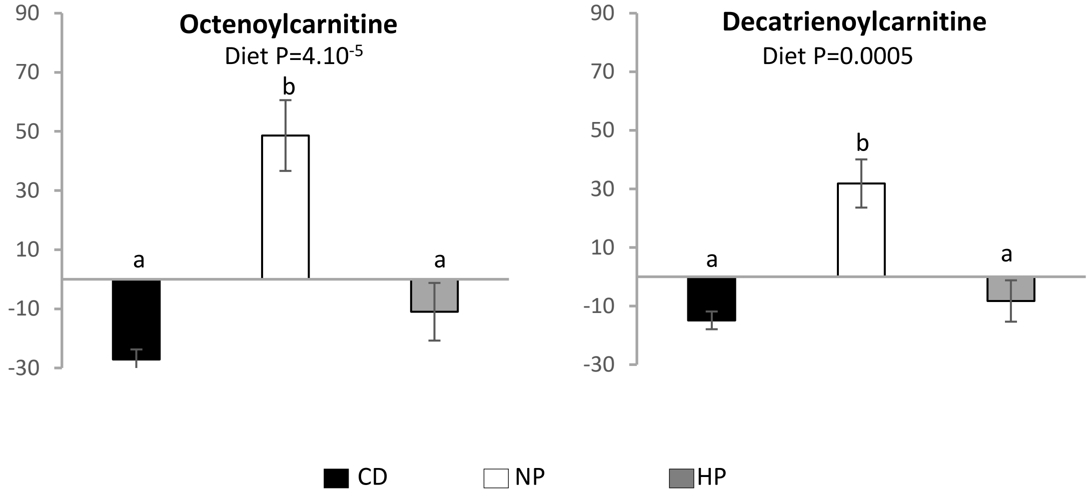

Octenoylcarnitine
Diet P=4.10-5
Decatrienoylcarnitine
Diet P=0.0005
90
70
50
30
10
-10
-30
a
b
a
CD
NP
HP

Figure 6

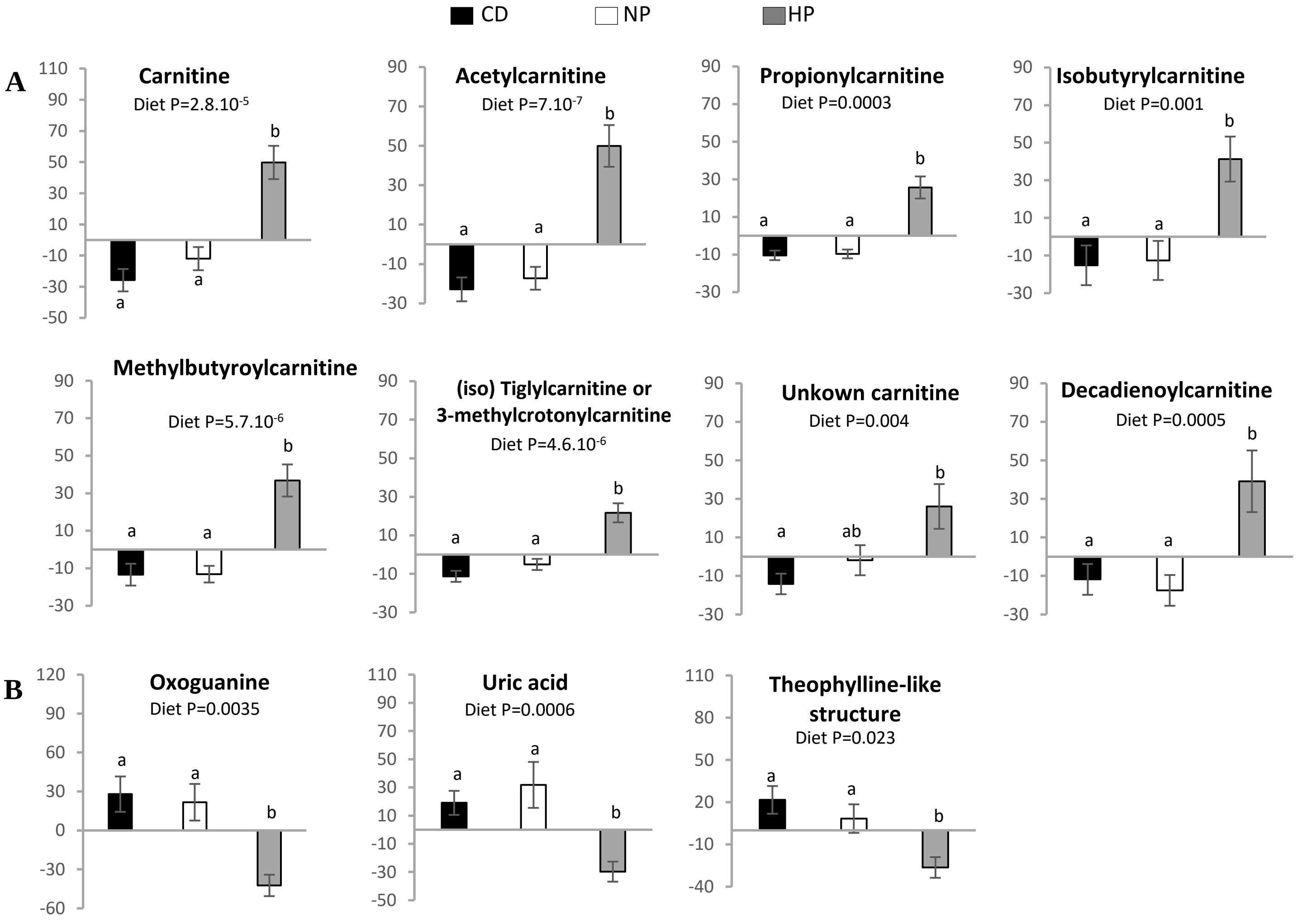
CD
NP
HP
A
Carnitine
Diet P=2.8.10-5
Acetylcarnitine
Diet P=7.10-7
Propionylcarnitine
Diet P=0.0003
Isobutyrylcarnitine
Diet P=0.001
Methylbutyroylcarnitine
Diet P=5.7.10-6
(iso) Tiglylcarnitine or 3-methylcrotonylcarnitine
Diet P=4.6.10-6
Unkown carnitine
Diet P=0.004
Decadienoylcarnitine
Diet P=0.0005
B
Oxoguanine
Diet P=0.0035
Uric acid
Diet P=0.0006
Theophylline-like structure
Diet P=0.023

Figure 7

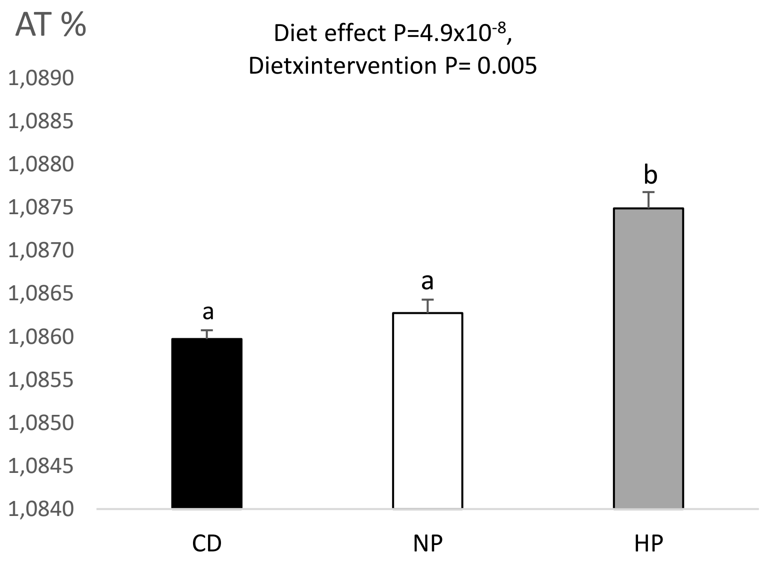
AT %
Diet effect P=4.9x10-8,
Dietxintervention P= 0.005
1,0890
1,0885
1,0880
1,0875
1,0870
1,0865
1,0860
1,0855
1,0850
1,0845
1,0840
a
a
b
CD
NP
HP

Figure S1: ^{13}C enrichment observed in 24h urines of participants, in all dietary groups; participants were adapted to either a CD for 4 weeks (n=10) or a HD differing in protein content, NP vs. HP in random order (NP-HP, n=8; HP-NP, n=9), P1 and P2. Data are mean ± SEM. The *P* value is indicated on graph, when the difference is to be significant, for the diet and period effect (ANOVA). When the effect is significant, different letters indicated significant differences between groups. CD, control diet; NP, Normal protein, high-fat, hypercaloric diet; HP, High protein, high-fat, hypercaloric diet.

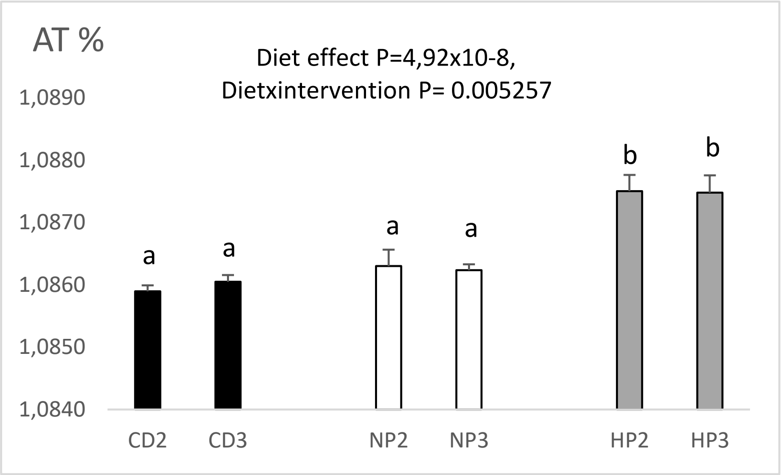

Figure S2. Identification of acetylcarnitine ($C_9H_{18}NO_4$). **A** MS-MS spectrum of the peak characterized by R_T 0.50 min, $[M+H]^+$= 204.1216 obtained with a collision trap energy = 15 eV; **B** – MS-MS spectrum of authentic standard of acetylcarnitine.

A

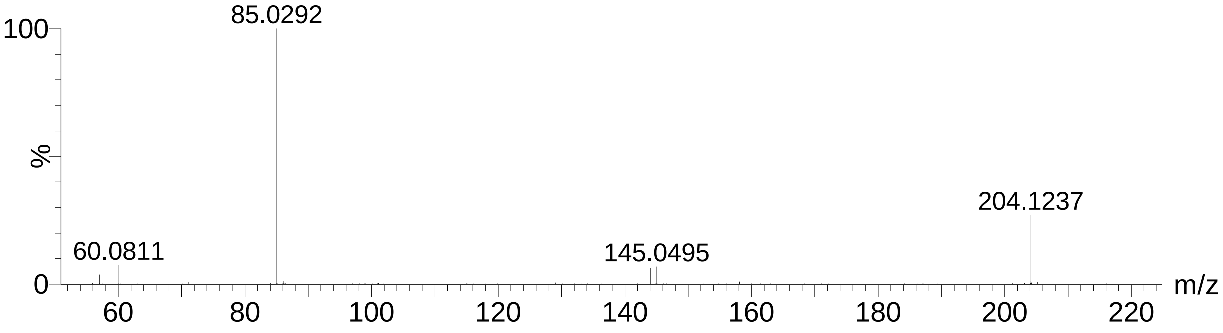

B

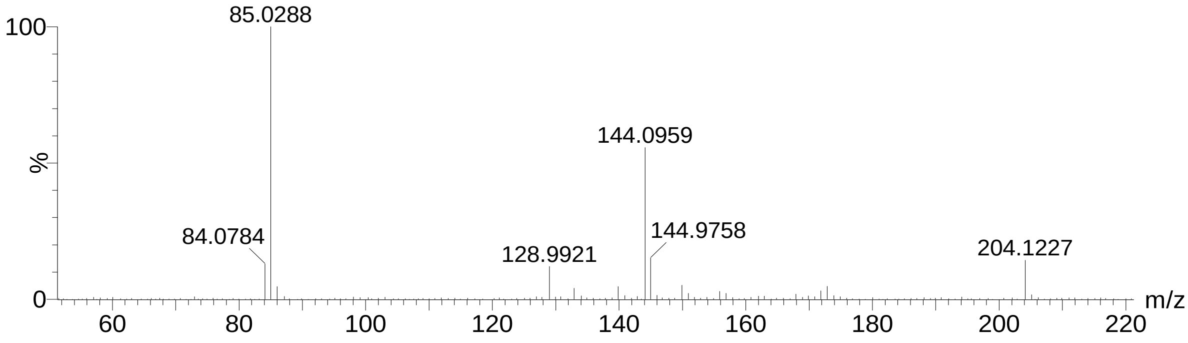

Figure S3. Identification of propionylcarnitine ($C_{10}H_{20}NO_4$). **A** MS-MS spectrum of the peak characterized by R_T 0.59 min, $[M+H]^+$= 218.1374 obtained with a collision trap energy = 15 eV; **B** – MS-MS spectrum of authentic standard of propionylcarnitine.

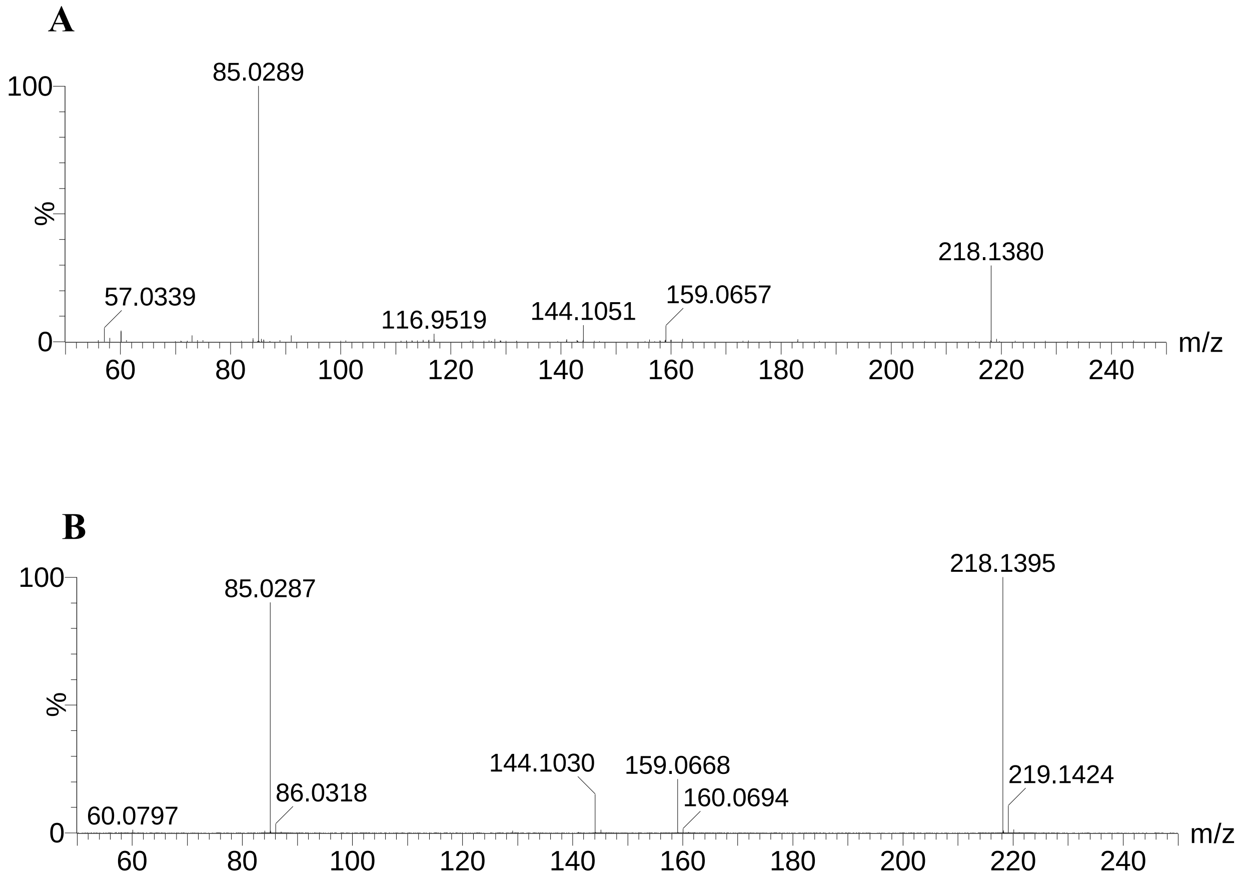

Figure S4. Identification of isobutyrylcarnitine ($C_{11}H_{22}NO_4$). **A** MS-MS spectrum of the peak characterized by R_T 0.70 min, $[M+H]^+$= 232.1529 obtained with a collision trap energy = 15 eV; **B** – MS-MS spectrum of authentic standard of isobutyrylcarnitine.

A

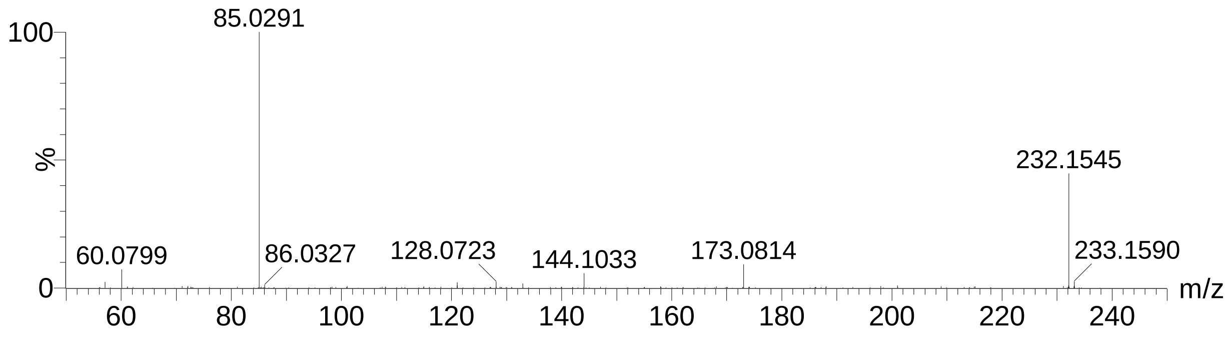

B

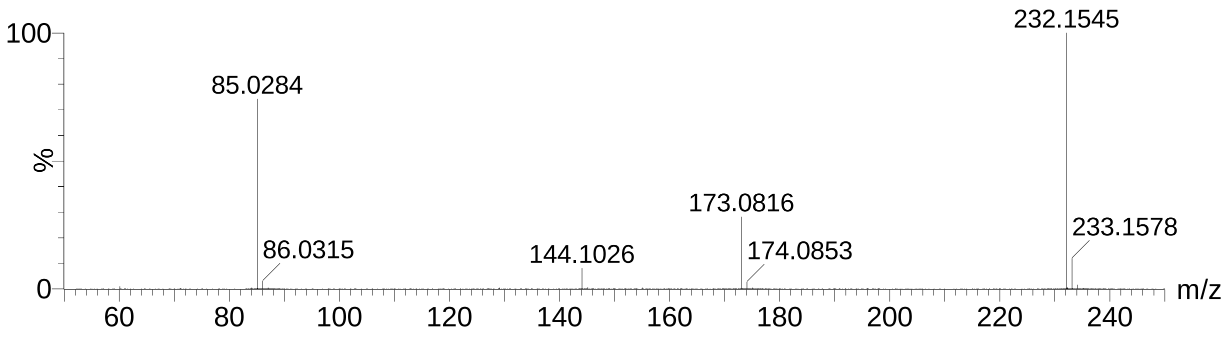

Figure S5. Identification of carnitine ($C_7H_{16}NO_3$). **A** – MS-MS spectrum of the peak characterized by R_T 0.48 min, $[M+H]^+$= 162.1114 obtained with a collision trap energy = 20 eV; **B** – MS-MS spectrum of authentic standard of carnitine.

A

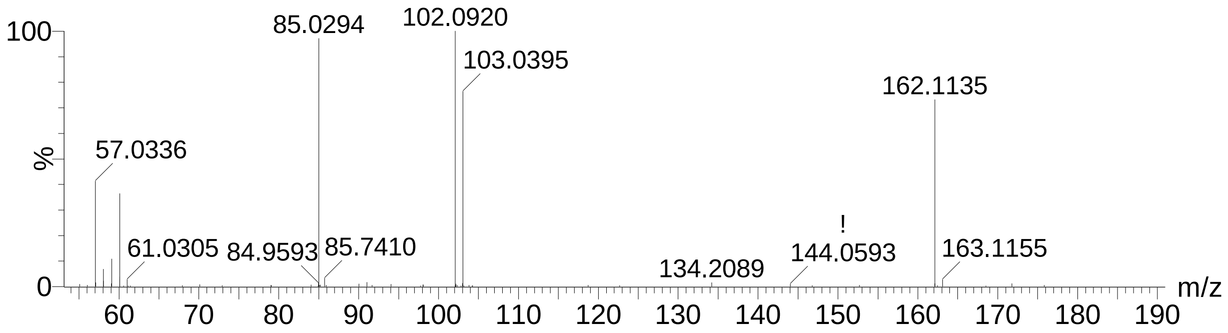

B

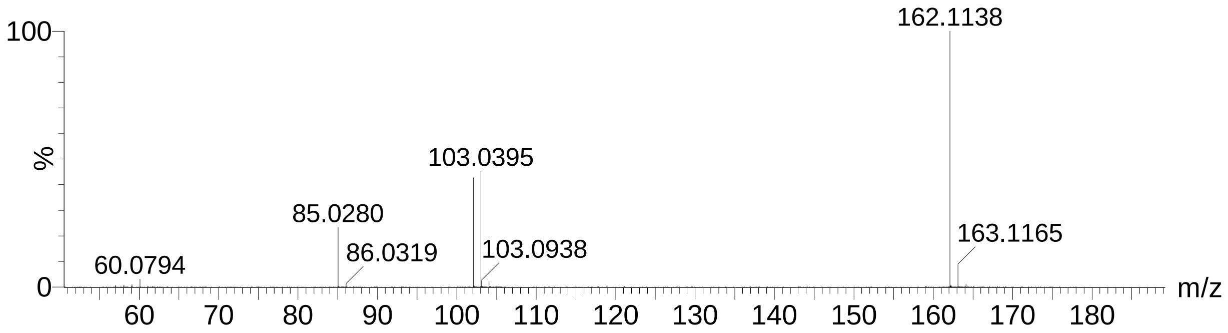

Figure S6. Identification of uric acid ($C_5H_5N_4O_3$). **A** – MS-MS spectrum of the peak characterized by R_T 0.66 min, $[M+H]^+$= 169.0355 obtained with a collision trap energy = 20 eV; **B** – MS-MS spectrum of authentic standard of uric acid.

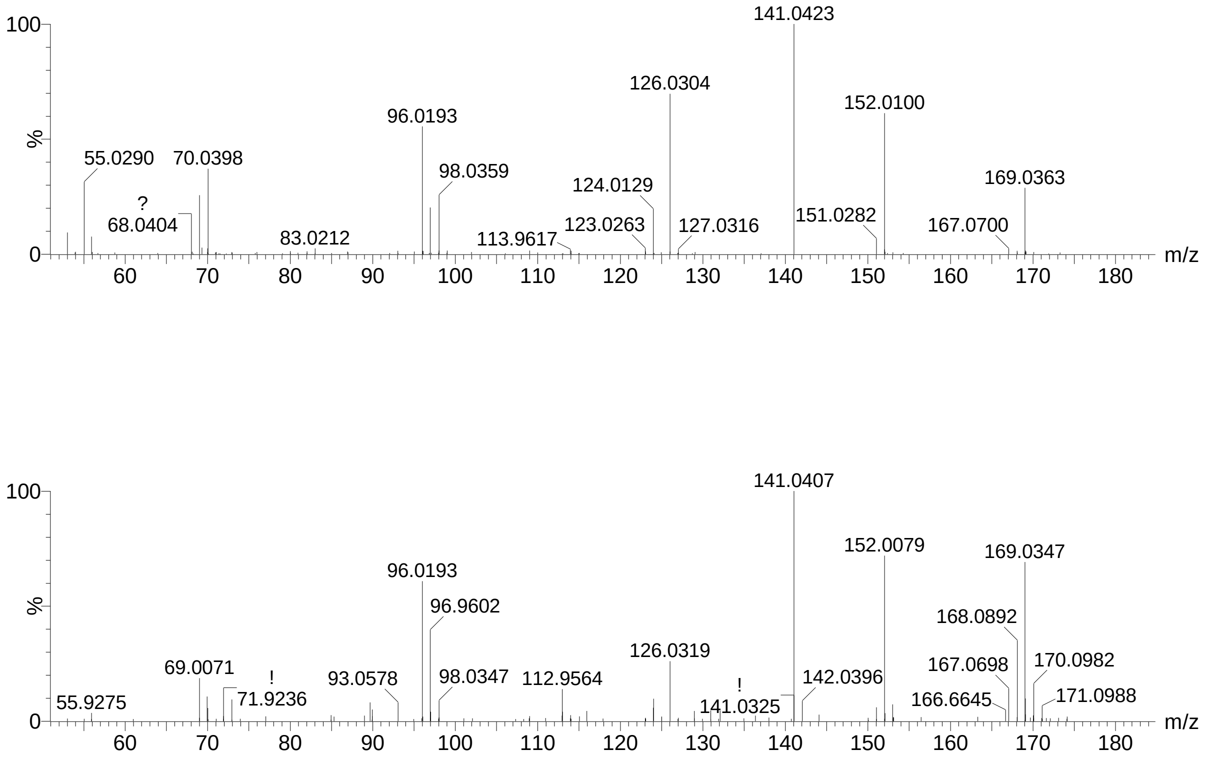

Supplement: Supplementary file 1 [file nutrients-13-01191-s001.zip › SUPPL/nutrients-1140815 FIGURE.pdf]
