# Supplementary material for: Urinary Medium-Chained Acyl-Carnitines Sign High Caloric Intake whereas Short-Chained Acyl-Carnitines Sign High -Protein Diet within a High-Fat, Hypercaloric Diet in a Randomized Crossover Design Dietary Trial
Source: Nutrients. 2021 Apr 3;13(4):1191. doi: 10.3390/nu13041191 (PMC8066704; doi:10.3390/nu13041191)
Supplement: Supplementary file 1 [file nutrients-13-01191-s001.zip › SUPPL/supplemental table S1.docx]

| R_T_, min (RP/HILIC) | Experimental  m/z | RP/HILIC | Elemental composition | MS/MS fragment ions | Collision energy, eV | MSI MI level | Annotation/identification |
| --- | --- | --- | --- | --- | --- | --- | --- |
| 0.47/0.90 | 114.0657 | RP/HILIC | C_4_H_8_N_3_O | 86.0716 | 20 | 2 | Creatinine |
| 0.53/3.81 | 132.0705 | RP/HILIC | C_4_H_10_N_3_O_2_ | 90.0559, 87.0725, 72.9370 | 15 | 2 | Creatine |
| 0.58/3.82 | 138.0544 | RP/HILIC | C_7_H_8_NO_2_ | 136.0407, 94.0656, 92.0476 | 30 | 3 | A structure similar to Methyl-nicotinamide |
| 0.59 | 365.1046 | RP | -- | No MS-MS | -- | -- | Unknown |
| 0.64/4.76 | 229.1536 | RP/HILIC | C_11_H_21_N_2_O_3_ | 142.0865, 96.0815, 70.0655 | 30 | -- | Unknown |
| 4.60 | 300.2171 | RP | C_16_H_30_NO_4_ | 241.1440, 144.1030, 139.1114, 121.1007, 111.1176, 85.0290 | 20 | 2 | (C9:1) carnitine |
| 4.99 | 302.2326 | RP | C_16_H_32_NO_4_ | 243.1518, 159.1384, 123.1161, 144.1022, 85.0291 | 15 | 2 | (Iso)nonanoylcarnitine or  2-methyloctanoylcarnitine |
| 6.17 | 288.2532 | RP | C_16_H_34_NO_3_ | 270.2406, 227.2026, 106.0869, 88.0760 | 20 | 2 | Lauroyl diethanolamide |
| 7.09 | 409.1626 | RP | -- | No MS-MS | -- | -- | Unknown |
| 7.09 | 387.1806 | RP | C_22_H_27_O_6_ | 371.2334, 325.4536, 233.0774, 189.1146, 105.0707 | 10 | -- | Unknown |
| 7.09 | 432.2385 | RP | -- | No MS-MS | -- | -- | Unknown |
| 0.42/1.42 | 170.0915 | RP/HILIC | C_7_H_12_N_3_O_2_ | 124.0852, 109.0742, 97.1008, 96.0728,  95.0658, 83.0575 | 20 | 1 | 1-Methylhistidine |
| 0.60/3.24 | 144.1014 | RP/HILIC | C_7_H_14_NO_2_ | No MS-MS | -- | -- | Unknown |
| 0.60/4.00 | 160.0962 | RP/HILIC | C_7_H_14_NO_3_ | No MS-MS | -- | -- | Unknown |
| 3.52/2.14 | 287.1005 | RP/HILIC | C_13_H_17_N_2_O_4_ | 147.0772, 136.0763, 130.0508, 129.0672, 91.0549, 84.0454 | 15 | 1 | Phenylacetylglutamine |
| 1.39 | 166.0731 | HILIC | C_6_H_8_N_5_O | 149.0469, 124.0513, 107.0256, 94.0430 | 20 | 1 | 7-Methylguanine |
| 2.81 | 282.12 | HILIC | C_11_H_16_N_5_O_4_ | 151.0780 | 15 | 2 | 1-Methyladenosine |
| 0.63 | 153.0665 | HILIC | C_7_H_9_N_2_O_2_ | 136.0392, 125.4551, 122.0228, 110.0632, 108.0448 | 20 | 3 | N-Methyl-2-pyridone-5-carboxamide |
| 0.44 | 137.0716 | HILIC | C_7_H_9_N_2_O | 120.0431, 94.0647, 92.0507, 79.0431, 65.0375 | 30 | 3 | 6-Methyl-pyridine-3-carboxamide |

**Table S1.** MS characteristics of diet-associated non-discriminant features detected by RP-chromatography and HILIC.

HILIC: hydrophilic interaction chromatography, MS: Mass spectrometry, MSI MI: level - Metabolomic standards initiative  metabolite identification level, RP: reverse-phase chromatography Rt: retention time
